# Supplementary material for: Quercetin’s Dual Mode of Action to Counteract the Sp1-miR-27a Axis in Colorectal Cancer Cells
Source: Antioxidants (Basel). 2023 Aug 2;12(8):1547. doi: 10.3390/antiox12081547 (PMC10451631; doi:10.3390/antiox12081547)
Supplement: Supplementary file 1 [file antioxidants-12-01547-s001.zip › antioxidants-2483040-supplementary.pdf]

Supplementary material

Quercetin’s dual mode of action to counteract the Sp1-miR-27a axis in colorectal cancer cells

Emanuele Fosso [†], Manuela Leo [†], Livio Muccillo, Vittorio Maria Mandrone,
Maria Chiara Di Meo, Annamaria Molinario, Ettore Varricchio, Lina Sabatino *

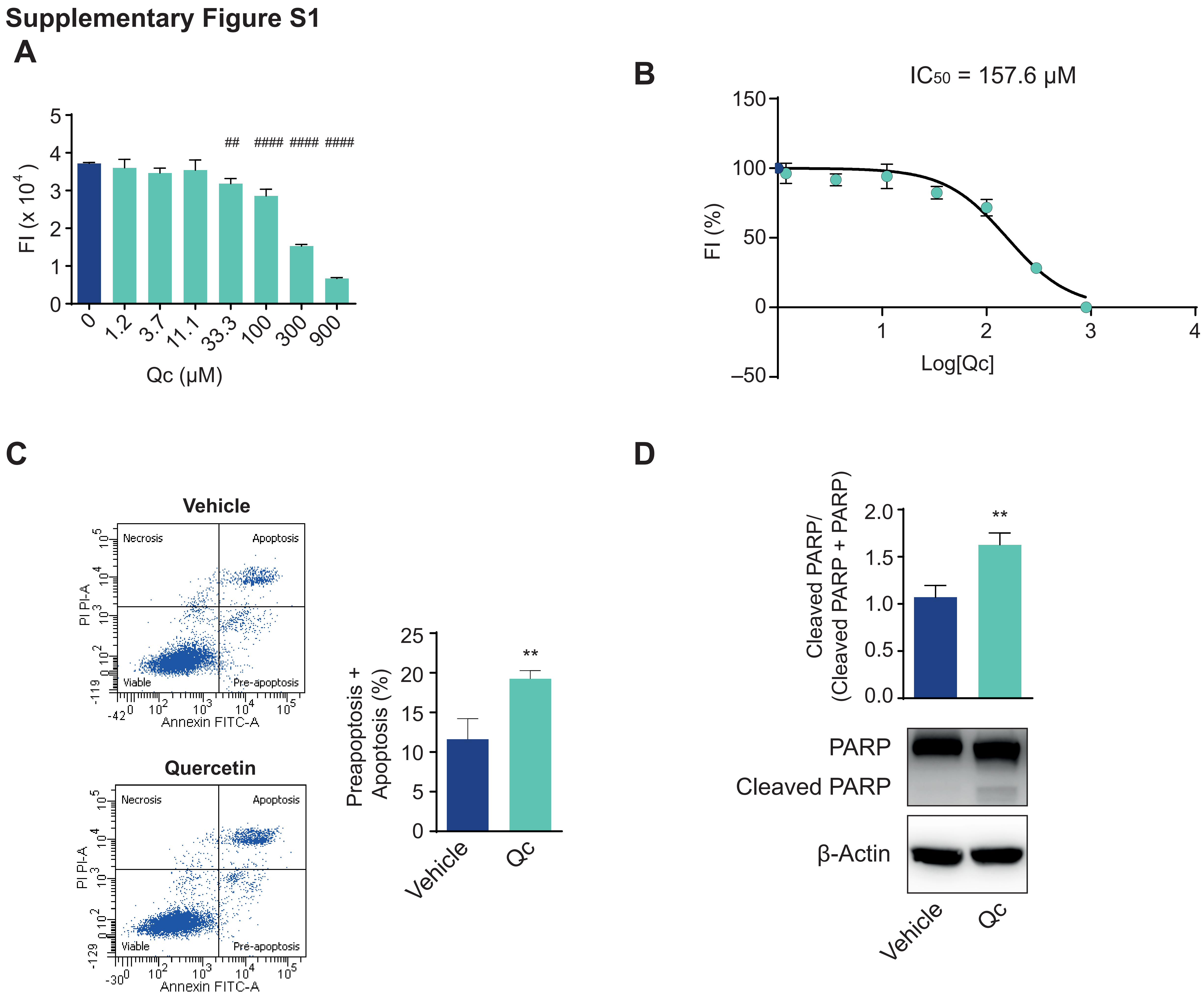

Supplementary Figure S1. Quercetin influences viability and apoptosis of HT-29 CRC cells
(**A**) Cell viability of HT-29 cells treated with increasing doses of Qc (from 0 to 900 µM) for 24 h assessed by FI. (**B**) Calculation of IC_{50} value in HT-29 cells treated with Qc as in (**A**). (**C**) A flow cytometry analysis of HT-29 cells treated with 150 µM Qc for 24 h and assayed with the annexin V/propidium iodide test. The histogram reports the percentages corresponding to the sum of pre and apoptotic cells.
(**D**) A western blot analysis of the cleaved form of PARP normalized to the total form in HT-29 cells treated as in (**C**) and referred to vehicle-treated cells. β-Actin is shown as loading control.
Statistical significance is considered when ## $p < 0.01$, and #### $p < 0.0001$ (ANOVA with Dunnett's post-test) or ** $p < 0.01$ (t-test).

Supplementary Figure S2

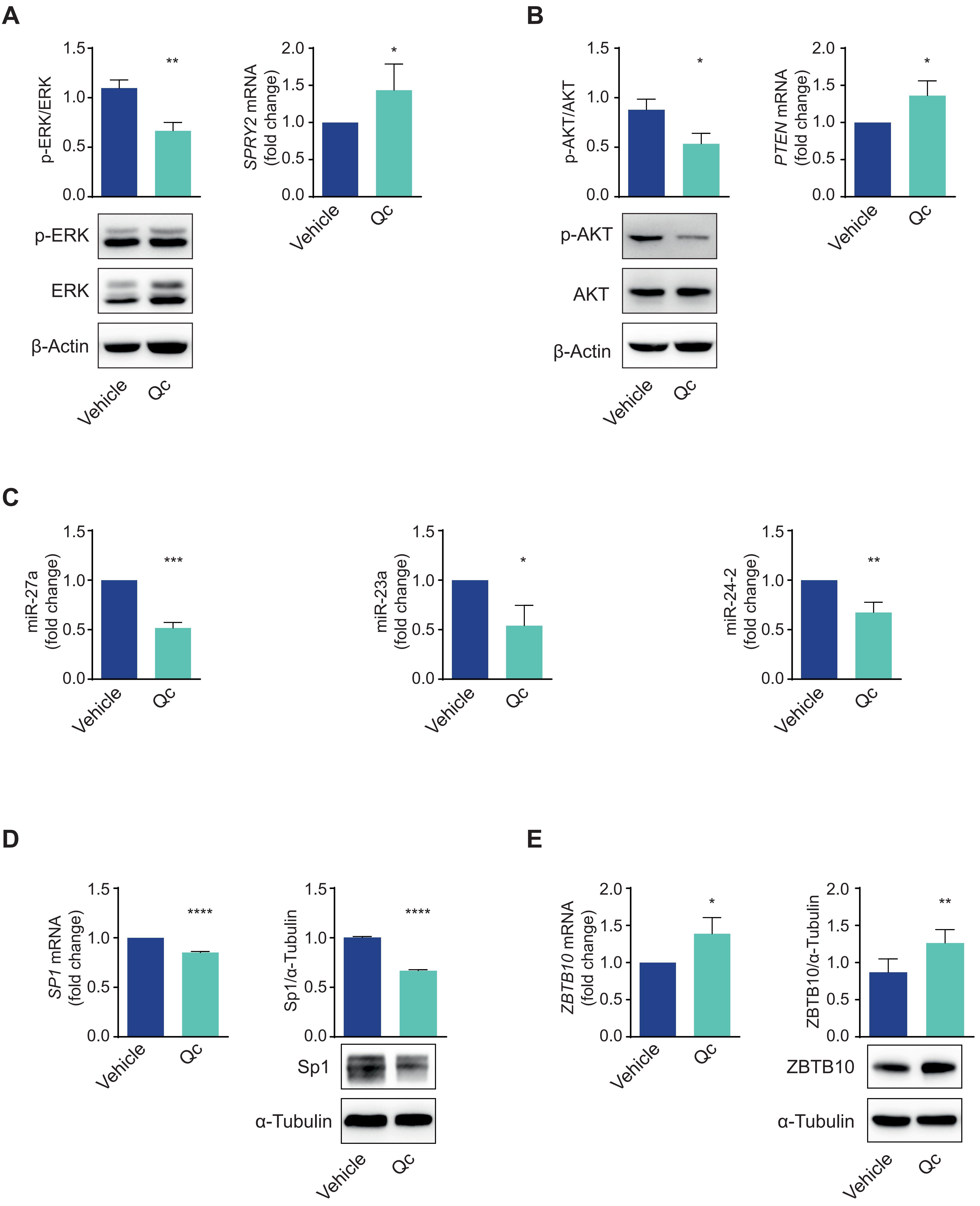

Supplementary Figure s2. Quercetin influences the same pathways observed/studied in HCT116 cells. (**A**) A western blot analysis of ERK phosphorylation (left panel) and a qRT-PCR of *SPRY2* (right panel) in HT-29 cells treated with 150 µM Qc for 24 h with respect to vehicle treated cells. (**B**) A western blot analysis of AKT phosphorylation (left panel) and qRT-PCR of *PTEN* (right panel) in HT-29 cells treated as in (**A**) compared to vehicle-treated cells. (**C**) a qRT-PCR of miR-27a, miR-23a, and miR24-2 in HT-29 cells treated as in (**A**) and compared to vehicle-treated cells. A western blot (left panel) and a qRT-PCR (right panel) analysis of (**D**) Sp1 and (**E**) ZBTB10 in HT-29 cells treated as above with respect to vehicle-treated cells.
Statistical significance is considered when * $p < 0.05$, ** $p < 0.01$, *** $p < 0.001$, and **** < 0.0001 (t-test).
